# Chemical and Biological Threats: Guidance for Breastfeeding Women, Infants, and Young Children

**DOI:** 10.1089/hs.2023.0096

**Published:** 2024-04-16

**Authors:** Sharon Leslie, Mija Ververs

**Affiliations:** Sharon Leslie, DPT, MPH, was a Research Associate; at the Center for Humanitarian Health, Johns Hopkins Bloomberg School of Public Health, Baltimore, MD.; Mija Ververs, MMed, MPH, RD, is a Senior Associate; at the Center for Humanitarian Health, Johns Hopkins Bloomberg School of Public Health, Baltimore, MD.; Sharon Leslie is now a Global Health Consultant, Los Altos, CA.

**Keywords:** CBRN, Breastfeeding, Chemical agents, Biological agents, Infant feeding, Emergency preparedness/response

## Introduction

In today's modern warfare, there is a growing risk of chemical and biological weapons use. Despite the widespread adoption of the United Nations Biological and Toxin Weapons Convention^1^ in 1975 and the Chemical Weapons Convention^2^ in 1997, we have seen notable examples of state-sponsored use of agents on vulnerable populations, as well as the use by individuals and nonstate actors.^3-7^ With the rise in acts of terrorism and conflict, there is an increased likelihood of exposure to chemical or biological agents by women and young children, who often represent the most vulnerable among the population. Children aged 0 to 5 years carry the most significant burden of conflict-related deaths of all age groups.^8^ Women of reproductive age who live in conflict zones have 3 times higher mortality than women who live in predominantly conflict-free settings.^9^

Despite the stark evidence of severe short- and long-term impacts of conflict on women and young children, this population has historically been overlooked when it comes to setting policy or providing holistic or human-centered guidance in conflict settings.^10^ There are abundant guidelines and research available on chemical and biological threats and how the general population could be impacted, as well as references to the treatment of breastfeeding women when discussing antidotes, vaccines, and drug treatments. However, there is very little guidance on whether women can safely continue to breastfeed after specific chemical and biological events and at what point breastfeeding can be safely resumed. Current information about breastfeeding safety in this scenario is disparate and hard to find.

As of 2023, there was no central repository of specific, easy-to-understand information for breastfeeding mothers and infants about the effects of chemical or biological agents. Guidelines written by normative agencies are typically agent-specific, and information about population or context-specific safety is ambiguous and not easy for first responders to readily access or act upon.^11-14^ It is vital to have clear and easy-to-access information about the safety of breastfeeding after exposure to a chemical or biological agent, whether and for how long to stop breastfeeding due to risks of transmission to the child, and how to best support women and children with alternative feeding options and education on relactation.

In response to the lack of critical information, the Johns Hopkins Center for Humanitarian Health, along with the Infant Feeding in Emergencies Core Group, created guidance for the breastfeeding population in the context of the most common chemical and biological agents.^15-17^ The development of this evidence-based guidance was a multidisciplinary effort, drawing on expertise from a wide variety of fields, including medical toxicology; infant feeding in emergencies; health security; chemical, biological, radiological, and nuclear (CBRN) preparedness; infectious disease; and disaster response. The guidance is intended for policymakers, healthcare workers, and emergency planners and can be used alongside existing guidance for the general public and healthcare workers.

## Importance of Guidance for Breastfeeding Women and Infants

Breastfeeding is known to save lives in emergencies and conflict situations by providing infants and young children with vital hydration, high-quality nutrition, comfort, warmth, and connection. Protection against disease and food security are critical during emergencies when there is often a lack of access to clean water, electricity, food supplies, and healthcare. Supply chains can also be affected in conflict settings, leading to difficulties in feeding infants dependent on commercial milk formula. Women also face challenges during emergencies in continuing breastfeeding due to the potential lack of support systems and lack of quality, easy-to-understand information about the safety of breastfeeding in the wake of a chemical or biological threat. A 2019 study in Iraq showed a negative association between breastfeeding status and exposure to conflict-associated circumstances.^18^ In Ukraine, in 2015, 46% of internally displaced women stopped breastfeeding children under the age of 6 months due to conflict-related situations and stress.^19^

Breastfeeding practices can be undermined by misinformation, inconsistencies in guidelines, and poorly informed responders during emergencies. The most recent example stems from the reaction to the COVID-19 pandemic. Despite guidance from the World Health Organization (WHO) and other international health agencies that explicitly supported breastfeeding with appropriate precautions for mothers with either confirmed or suspected COVID-19, recommendations against breastfeeding were common across the world and were slow to change, even as it became clear that COVID-19 was not transmitted through breastmilk.^20,21^ The misinformation and inconsistency of advice sowed confusion and mothers were often told to weigh the risks and benefits and make their own individual decisions.^21^

In the event of a chemical or biological attack, infants and young children present a unique set of challenges—not only must they be fed every few hours, but they also have greater risks of exposure compared with adults in terms of both physiological and anatomical perspectives.^22^ Children have a higher ratio of skin surface area to mass, a faster respiratory rate, and increased skin permeability, making them more vulnerable to the impact of biological and chemical agents.^23^ Decisions for this population—and for breastfeeding mothers to whom infants are dependent on for survival—are therefore different than for the general population. Information must be easily accessible and readily actionable, and it must be integrated into existing guidelines to increase the chance of saving lives during a chemical or biological attack.

## Methodology

The impetus for this study came at the beginning of the war in Ukraine when bombing occurred near several nuclear power plants. While we began our research by looking at how to best protect breastfeeding women and infants in light of a nuclear power plant emergency, it quickly became clear that expanding the scope to chemical and biological threats was critically necessary given the state of modern warfare. Further, it was evident that CBRN guidance for women and children was relevant and important for all conflicts, not just Ukraine. Therefore, our work expanded to the full scope of CBRN as it relates to breastfeeding safety during times of war or conflict throughout the world. Please note that this commentary addresses only the process specific to chemical and biological agents.

We began by conducting a literature review, covering the period from January 1990 to October 2022, of available studies and published guidelines on chemical and biological agents, their mechanism of action, and the approved treatment. A further review was conducted on breastfeeding safety in emergencies, specific recommendations, and precautions associated with both the agents and treatments as they relate to breastfeeding and infant and young child feeding. We analyzed data on chemical and biological agents from organizations including WHO, the US Centers for Disease Control and Prevention, the European Centers for Disease Control, the US Food and Drug Administration, and the US National Institutes of Health. In addition, we consulted with experts in CBRN threats, chemical weapons, toxicology, and health security; experts in infant and young child feeding in emergencies, and breastfeeding; and representatives from the US Centers for Disease Control and Prevention and the US Food and Drug Administration. Following the final approval of guidance by the Infant Feeding in Emergencies Core Group and the research team at the Johns Hopkins Center for Humanitarian Health, draft fact sheets and guidance were sent to an international group of expert reviewers for feedback, comment, and subsequent revision.*

## Scope of Guidance

Context matters when evaluating breastfeeding safety after exposure to a chemical or biological agent. In wartime or crisis situations, there is often limited access to alternative feeding options and time is critical for both the health of the mother and child. When creating the guidance, we weighed several different factors that can impact breastfeeding safety. These included the known short- and long-term benefits of breastfeeding for the infant; the duration, level, and route of exposure of the chemical or biological agent for the mother and infant, which may or may not be known; the immediate need for medication for the mother; the level of contagion and potential transmission of the agent to the child; the potential impact of medication or antidote used for treatment on milk production; the amount of medication or antidote excretion into breastmilk; and the potential adverse effects on the breastfeeding infant. The expertise and collaboration of CBRN experts, toxicologists, infectious disease experts, and infant nutrition experts was critical for us to fully integrate all considerations into comprehensive guidance for each agent.

The guidance notes outline the safety of common antimicrobials and treatments and breastfeeding.^16,17^ Note that while Tables 1 and 2 serve as brief summaries and quick references of the information contained in the guidance notes, given the complexity of each agent, a greater level of detail about management and breastfeeding safety is given in the full guidance notes, which policymakers and healthcare providers can use to make decisions.

**Table 1.** Summary of Breastfeeding Safety and Treatment by Chemical Agent

Agent	Treatment for Breastfeeding Women	Treatment for Infants and Children	Is Breastfeeding Safe After Exposure?	Is Breastfeeding Safe During Treatment?
Chlorine gas	Decontamination, supportive care, and treatment of pulmonary injury^24^ Treatment: albuterol, sodium bicarbonate, prednisone, prednisolone^14,24^	Decontamination, supportive care, and treatment of pulmonary injury^24^ Treatment: nebulized sodium bicarbonate, inhalation of albuterol via metered-dose aerosol.^25^	Yes. Breastfeeding can continue if the mother is physically able to do so.	Yes. Breastfeeding can continue if the mother is physically able to do so. Albuterol and sodium bicarbonate are considered safe for breastfeeding women.^26^ Prednisone and prednisolone are considered safe with breastfeeding.^27-29^
Hydrogen cyanide	Decontamination, supportive care^30^ Antidotes: IV hydroxocobalamin, nithiodote (sodium nitrite with sodium thiosulfate), amyl nitrite^31,32^	Decontamination, supportive care^31^ Antidotes: IV hydroxocobalamin, nithiodote (sodium nitrite with sodium thiosulfate) (not for infants under 6 months of age)^32^ dosing is available for the pediatric population	No. Temporarily interrupt for 15 days postexposure.^33^	No. Temporarily interrupt for 15 days postexposure.^33^ If a breastfeeding mother is treated with nithiodote (sodium nitrite with sodium thiosulfate), breastfeeding should be temporarily interrupted.^32^ IV hydroxocobalamin (vitamin B12) is considered safe for breastfeeding women.^27,28^
Lewisite	Decontamination, supportive care^35^ Antidote: intramuscular injection of British anti-Lewisite (BAL; also called dimercaprol); due to its significant side effects, it is recommended only for people who have signs of shock or significant pulmonary injury^35,36^ Contraindicated in anyone with a peanut allergy	Decontamination, supportive care^34^ Antidote: intramuscular injection of BAL; due to its significant side effects, it is recommended only for people who have signs of shock or significant pulmonary injury^35,36^; dosing is available for the pediatric population but not for the infant population^37^	No. Breastfeeding should be temporarily interrupted. Arsenic can be excreted into breastmilk and can be toxic to an infant.^38^ Can resume after 15 days if the mother is physically able.^36,37,39^	No. Can resume after 15 days if the mother is physically able to do so with or without treatment.^36,37,39^ (See the full recommendation for lewisite for information on the half-life of both lewisite and BAL in the guidance note) BAL is considered contraindicated by some sources for breastfeeding women given its possible excretion into breastmilk.^37^
Sarin	Decontamination, supportive care^13^ Antidote: atropine and pralidoxime chloride (2-PAM); diazepam or midazolam when there is evidence of seizures^13^	Decontamination, supportive care^13^ Antidote: atropine and 2-PAM if >1 year of age, only atropine if <1 year of age; diazepam when there is evidence of seizures^13^	No. Breastfeeding should be halted. In the absence of knowledge and data, if a safe breastmilk substitute (BMS) alternative is available, halt breastfeeding.^40,41^	No. Breastfeeding should be halted. In the absence of knowledge and data, if a safe BMS alternative is available, halt breastfeeding.^40^
Sulfur mustard	Decontamination, supportive care^42^ Pain medication for eye pain, blisters, skin burns; oral antihistamines for skin itching and irritation	Decontamination, supportive care^42^ Pain medication and oral antihistamines	No. Breastfeeding should be halted. Halt breastfeeding due to the high fat solubility of sulfur mustard.^43^	No. Breastfeeding should be halted. Halt breastfeeding due to the high fat solubility of sulfur mustard.^43^
Tabun	Decontamination, supportive care^44^ Antidote: atropine and 2-PAM; diazepam when there is evidence of seizures^44^	Decontamination, supportive care^44^ Antidote: atropine and 2-PAM if over 1 year of age, only atropine if under 1 year of age; diazepam when there is evidence of seizures^44^	No. Breastfeeding should be halted. In the absence of knowledge and data, if a safe BMS alternative is available, halt breastfeeding.^40,41^	No. Breastfeeding should be halted. In the absence of knowledge and data, if a safe BMS alternative is available, halt breastfeeding.^40,41^
VX	Decontamination, supportive care^45^ Antidote: atropine and 2-PAM; diazepam when there is evidence of seizures^45^	Decontamination, supportive care^45^ Antidote: atropine and 2-PAM if >1 year of age, only atropine if <1 year of age; diazepam when there is evidence of seizures^45^	No. Breastfeeding should be halted. In the absence of knowledge and data, if a safe BMS alternative is available, halt breastfeeding.^40,41^	No. Breastfeeding should be halted. In the absence of knowledge and data, if a safe BMS alternative is available, halt breastfeeding.^40,41^

Note: If there are clear guidelines on when breastfeeding can be resumed, the guidance states, “temporarily interrupt” and will give recommendations regarding when it is safe to resume. If there are no evidence-based guidelines on when to resume, the guidance uses the word “halt.”

Abbreviations: 2-PAM, pralidoxime chloride; BAL, British anti-Lewisite; BMS, breastmilk substitute; IV, intravenous.

**Table 2.** Summary of Breastfeeding Safety and Treatment by Biological Agent

Agent/Disease	Spread (Person to Person)	Treatment for Breastfeeding Women	Treatment for Infants and Young Children	Is Breastfeeding Safe After Exposure?	Is Breastfeeding Safe During Treatment?
Anthrax^a^	No	Antibiotics, firstline treatment: ciprofloxacin, levofloxacin, moxifloxacin; amoxicillin if the strain is susceptible to penicillin^46^	Antibiotics: ciprofloxacin, doxycycline; amoxicillin if the strain is susceptible to penicillin^46^	Yes. Breastfeeding is safe to continue after exposure.^47^ Women with active skin lesions from anthrax on the breast should avoid infant contact with the affected breast and not breastfeed from that breast until 48 hours after appropriate antimicrobial therapy has been initiated.^47^ Expressed breastmilk can be used safely if hygiene and protective precautions were taken during expression, including handwashing and ensuring that no lesions come in contact with pump equipment if using a pump.	Yes. Breastfeeding is safe to continue during treatment.^47^
Botulism^a^	No	Supportive care (including assisted breathing using a ventilator for breathing difficulties and IV fluids if the person cannot swallow); early IV administration of botulinum antitoxin heptavalent (HBAT)^48^	Supportive care and early IV administration of HBAT^48^ FDA-approved HBAT dose for infants (<1 year of age) is 10% of the adult dose, regardless of weight; HBAT dose for children (ages 1 to 16 years) is 20% to 100% of the adult dose^49^	While current evidence is limited, the evidence that exists suggests that breastfeeding by an infected mother is not harmful to her infant.^50^ It is unlikely that botulinum toxin enters breastmilk because of its large molecular weight.^50^ Infants of breastfeeding mothers should be monitored closely for signs and symptoms of botulism such as low muscle tone, a weak cry, gastrointestinal symptoms, or difficulty feeding and swallowing.^50^	Yes. Breastfeeding can continue while receiving the antitoxin HBAT.^49,50^ Infants of breastfeeding mothers should be monitored closely for signs and symptoms of adverse impacts from botulinum antitoxin if given to the mother and/or infant including flulike symptoms, such as fevers, chills, and malaise.^50^
Ebola^a^	Via fluids	Supportive treatment; vaccine if exposed and not yet symptomatic^51^ Breastfeeding women infected with EVD (or those with high-risk exposures) can also receive Ebanga and Inmazeb^52^	Supportive treatment	No. Breastfeeding should be halted if EVD is confirmed in either mother or child or if either one has confirmed exposure.^51^ Expressed breastmilk should be discarded.^51^ If mother and child with potential exposure and no symptoms, breastfeeding infants under 6 months may be considered if no alternative options are available.^51^ Following recovery from EVD, if a woman wants to resume breastfeeding, she should wait for 2 consecutive negative EVD breastmilk tests (separated by 24 hours) before resuming.^51^	No.
Plague^a^	Yes	Antibiotics: ciprofloxacin, streptomycin, levofloxacin, moxifloxacin, gentamicin, and doxycycline^28,53^; chloramphenicol is also considered effective but has the potential for serious adverse reactions in infants, so other drugs should be used preferentially for breastfeeding mothers^53^	Antibiotics for children >1 month of age: gentamicin, streptomycin, ciprofloxacin, and levofloxacin^53^	No. Breastfeeding should be temporarily interrupted until both mother and infant receive antimicrobial treatment or PEP.^53^	Yes. If a mother and infant are both receiving antimicrobial treatment or postexposure prophylaxis (PEP), then a mother with pneumonic plague may continue to breastfeed safely.^53^ If an infant does not receive antimicrobial treatment or PEP at the same time as the mother, the mother with pneumonic plague should temporarily interrupt breastfeeding until she has received 48 hours of antimicrobial treatment.^53^
Q Fever^b^	No	Antibiotic: doxycycline^54^	Antibiotic: doxycycline^54^	Yes. Breastfeeding is safe to continue after exposure.^55^	Yes. Breastfeeding is safe to continue during treatment.^55^
Smallpox^a^	Yes	Supportive treatment; vaccine if exposed and not yet symptomatic^56^	Supportive treatment	If the mother or infant is a confirmed case of smallpox and has symptoms, halt breastfeeding.^57^ Because people cannot transmit infection until they begin to show symptoms of fever and rash, personal contact between the mother and infant can continue, including breastfeeding, until the onset of fever.^57^ If either the mother or infant develops a fever, immediate isolation should begin.^57^	If a mother is exposed to smallpox but does not yet show any symptoms, she may receive a vaccine (JYNNEOS is recommended for breastfeeding women) to prevent smallpox infection.^58^ Once the mother receives the vaccine, breastfeeding can continue with proper infection control to avoid contact with the vaccine site. The mother should temporarily halt breastfeeding from that breast if breast lesions are present.^59^
Tularemia^a^	No	Antibiotics: streptomycin, gentamicin, doxycycline, and ciprofloxacin^59^	Antibiotics: gentamicin, doxycycline, ciprofloxacin^59^	Yes. Breastfeeding is safe to continue after exposure.	Yes. Breastfeeding is safe to continue during treatment.^27^

Note: If there are clear guidelines on when breastfeeding can be resumed, the guidance states, “temporarily interrupt” and will give recommendations regarding when it is safe to resume. If there are no evidence-based guidelines on when to resume, the guidance uses the word “halt.”

^a^ Category A bioterrorism agents/diseases – easy to disseminate or transmit; ^b^Category B bioterrorism agents/diseases – moderately easy to disseminate.^60^

Abbreviations: EVD, Ebola virus disease; FDA, US Food and Drug Administration; HBAT, botulinum antitoxin heptavalent; IV, intravenous; PEP, postexposure prophylaxis.

### Chemical Agents

As we compiled the guidance for chemical agents, we took into account that in chemical attacks, often medical measures must be administered rapidly and patients may not be conscious. Therefore, medical professionals may not be aware of the breastfeeding status of a patient. Additionally, because of the dearth of data on some chemical agents and breastfeeding safety in normative literature, decisions about whether to continue to breastfeed following a chemical attack may need to be made on a case-by-case basis. Having clear guidance on hand for first responders is crucial to provide more clarity and direction to those on the ground.

The chemical agents included in the guidance published were compiled based on recommendations from CBRN experts. These agents were either (1) most likely to be used in a chemical attack or (2) considered to have significant implications for the breastfeeding population if used. For example, while lewisite has become a less likely agent to be used in wartime or terrorism because it contains arsenic, the authors of the guidance felt it was important to include in the guidance, given the clear toxicity of arsenic for breastfeeding infants. Other agents were chosen for their clear risk of use in conflict situations like the Syrian civil war, where more than 336 chemical weapons attacks occurred between 2012 and 2022.^3^

We used the following common categories to group our guidance on chemical agents in order to ease integration into existing guidelines for both normative agencies and policymakers: nerve agents, pulmonary/choking agents, blood/systemic agents, and blistering agents.^31^ Within each category, there is a different mechanism of action, different medical management and fatality rates, and therefore a different impact on infant and young child feeding.

The guidance note on chemical agents covers key characteristics, medical management, and breastfeeding safety associated with the following agents: sarin, tabun, VX, chlorine, hydrogen cyanide, sulfur mustard, and lewisite.^16^ To provide as much clarity as possible, we outlined the most current information available on the safety of treatments used for each agent and current literature on whether the chemical agent in question can be excreted in breastmilk (Table 1).^16,17^

### Biological Agents

In the event of a biological agent attack on a population, large numbers of people may be impacted. In contrast with a chemical agent, a biological agent may not have an immediate impact because of the delay between exposure and illness, creating a challenge for medical responders.^61^ Biological agents with a high contagion rate can spread extensively from the original area of concern, creating further strain on the medical system.^60^ Some potential agents that may be used in a biological attack are anthrax, botulism, plague, Ebola, tularemia, Q fever, and smallpox.^17^ The agents we chose to include in the guidance note on biological agents represent all Category A biological agents, with the addition of some Category B agents that were considered a priority to discuss in the context of breastfeeding safety (Table 2).^60^

For each agent, we incorporated the level of contagion and specified whether the agent can spread person-to-person and how that impacts the safety of the breastfeeding mother and young child. In some cases, breastmilk may be safe, but the act of breastfeeding is not safe due to the nature of contagion of a specific biological agent. For example, because there is no evidence that anthrax can be transmitted through breastmilk, breastfeeding is safe to continue after exposure. However, if a woman has active lesions from anthrax on her breast, then infants should avoid contact with the affected breast and not breastfeed until 48 hours after appropriate antimicrobial therapy has been initiated.^47^

Expressed breastmilk can be used safely if hygiene and protective precautions were taken during expression, including handwashing and ensuring that no lesions came in contact with pump equipment if a pump was used.^47^ In these cases, it is important that breastfeeding mothers get the support and education they need to express breastmilk for their infant in a manner that maintains protective precautions. To that end, we provided resources and links in the appendix of the guidance note on maintaining breastmilk supply in the event of temporary disruption of breastfeeding.^17^

## What We Learned

The process of investigating and formulating guidance for the breastfeeding population in the face of chemical or biological threats uncovered several crucial insights for our team. The existing guidance in the literature focused primarily on ideal contexts rather than conflict settings, which means they often did not account for the complexity of conflict zones. Developing guidance for conflict settings requires thinking about compromises and alternative scenarios that might not be ideal. For example, if there are supply chain issues or physical movement restrictions, there may be limited or no access to alternative feeding options or the necessary medications or antidotes. If there is no access to safe water supply, alternative feeding options may not be safe to use. Decisions need to be made quickly for medical reasons, and because infants cannot wait hours to be fed. Even if we are able to clearly state the known risks, people on the ground may have to make decisions and compromises based on the information available to them about the specific context. Making this information easily available to first responders is, therefore, of critical importance so they can make educated decisions in times of crisis.

We also found that the areas of knowledge necessary to create evidence-based guidance were siloed, or separated by discipline. Some of the other disciplines we worked with had not collaborated on projects and had very different networks from which to draw expertise. We found there was great enthusiasm and willingness to work together on this project, leading to generative conversations across disciplines and opening up avenues for future collaboration. We urge the research and medical community to keep finding ways to continue to bridge the siloes between disciplines for the greater benefit of the people who are meant to be served by the important work.

## Future Research Directions

The guidance we developed reflects our collective knowledge and draws on expertise from relevant disciplines. There is currently a lack of information in the literature regarding breastfeeding and many of the agents considered most likely to be used in wartime or a terrorist attack. More multidisciplinary research is required to fill the knowledge gap to protect the most vulnerable people in our global population.

As modern warfare progresses, new agents may be used, necessitating updates to the guidance. We acknowledge that the guidance will evolve as more information becomes available and we consider the guidance notes to be living documents. We plan to revisit the guidance and add known and novel agents as needed. We currently have the following agents on the list to be considered in future guidance: phosgene, ricin, adamsite, toxic industrial chemicals, and Novichok nerve agents.

## Conclusion

The complexity of responding to a biological or chemical attack in a conflict situation is fraught for the general population, and even more so for vulnerable populations, such as breastfeeding women and infants. We created the guidance as scaffolding for disaster management agencies and policymakers to use in the context of their own countries or areas of focus, as well as for normative agencies to deepen their existing knowledge base by integrating the agent-specific approach with a population-centered approach looking at women and young children. The goal is for the guidance to serve as a basis for more timely research into the impact of CBRN threats on women and children, especially threats from novel agents. We created several guidance documents for dissemination, including a full CBRN package, which includes information regarding breastfeeding safety after a nuclear power plant disaster in times of war, as well as separate documents for each area of focus to best serve specific specialties.^15^ (Links to the full documents are available at https://www.ennonline.net/cbrn-iycfe.)

It is crucial that emergency guidelines provide caregivers with clear and accurate information, reassurance, and guidance, in order to protect, promote, and support appropriate infant and young child feeding in the event of a CBRN emergency. To best serve those in need, this vital information needs to be shared throughout the CBRN and emergency response community to ensure that its existence is known before an emergency occurs, especially in times of war. In the event of a chemical or biological attack, time is critical for all victims—and even more so for young infants and children as the most vulnerable among us. As the nature of armed conflict changes around the world, we should focus our priorities on the people most impacted and continue keeping our attention on this issue. Chemical and biological warfare is not a theoretical situation—it is a very real threat, and we need to be prepared to maximize our ability to save lives.

## Notes

^*^
Please refer to the acknowledgments section for a specific list of agencies and individuals represented.
